# The Controversial Role of Human Gut Lachnospiraceae

**DOI:** 10.3390/microorganisms8040573

**Published:** 2020-04-15

**Authors:** Mirco Vacca, Giuseppe Celano, Francesco Maria Calabrese, Piero Portincasa, Marco Gobbetti, Maria De Angelis

**Affiliations:** 1 Department of Soil, Plant and Food Sciences, University of Bari Aldo Moro, 70126 Bari, Italy; mirco.vacca@uniba.it (M.V.); francesco.calabrese@uniba.it (F.M.C.); maria.deangelis@uniba.it (M.D.A.); 2 Clinica Medica “A. Murri”, Department of Biomedical Sciences and Human Oncology, University of Bari Medical School, 70121 Bari, Italy; 3 Faculty of Science and Technology, Free University of Bozen, 39100 Bolzano, Italy; marco.gobbetti@unibz.it

**Keywords:** Lachnospiraceae, gut microbiota, gut microbial pathways, gut microbial metabolites, health, disease

## Abstract

The complex polymicrobial composition of human gut microbiota plays a key role in health and disease. Lachnospiraceae belong to the core of gut microbiota, colonizing the intestinal lumen from birth and increasing, in terms of species richness and their relative abundances during the host’s life. Although, members of Lachnospiraceae are among the main producers of short-chain fatty acids, different taxa of Lachnospiraceae are also associated with different intra- and extraintestinal diseases. Their impact on the host physiology is often inconsistent across different studies. Here, we discuss changes in Lachnospiraceae abundances according to health and disease. With the aim of harnessing Lachnospiraceae to promote human health, we also analyze how nutrients from the host diet can influence their growth and how their metabolites can, in turn, influence host physiology.

## 1. Introduction

**Figure 1:**
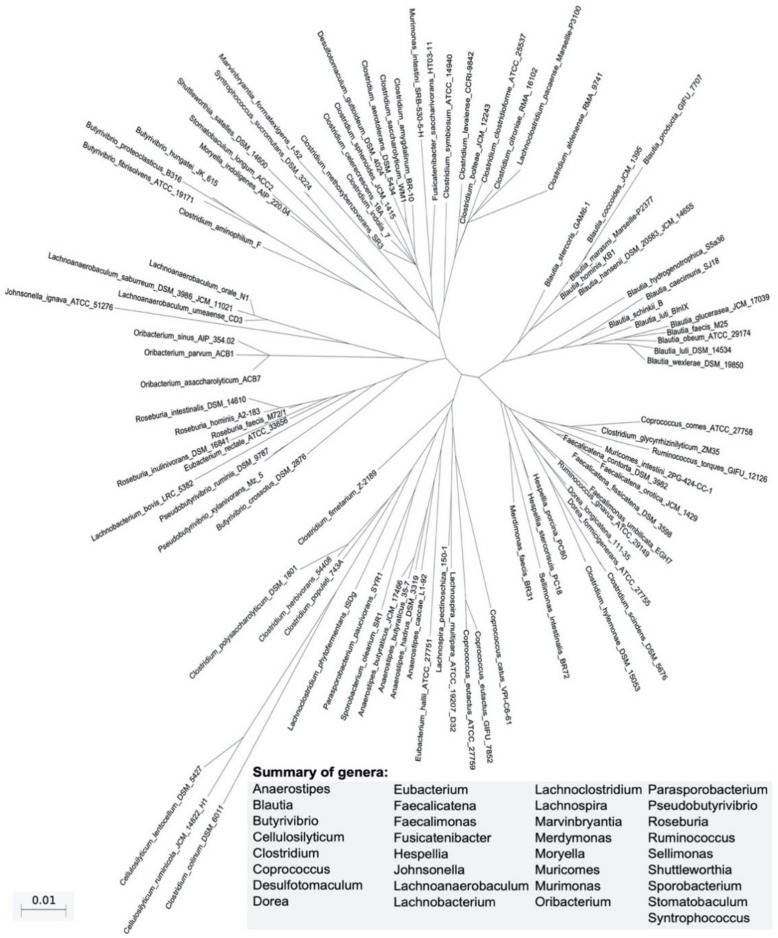
Phylogeny of taxa belonging to the Lachnospiraceae family. Sequences have been retrieved from the RefSeq Targeted Loci Project included in the National Center for Biotechnology Information (NCBI) database using the following combined search: txid186803[ORGN] AND (33175[Bioproject] OR 33317[Bioproject] of bacterial 16S ribosomal RNA. The nucleotide sequences have been multiply aligned using MAFFT tool version 7.427 (https://mafft.cbrc.jp/alignment/software/) and the approximately-maximum-likelihood phylogenetic tree has been inferred from the nucleotide alignments by using the general time-reversible model (GTR).

The human gastrointestinal (GI) tract has an estimated surface of more than 200 square meters and represents the interface between the body and the external environment, hosting a complex polymicrobial ecology that includes bacteria, archaea, fungi, protists, and viruses. The population of human gut microorganisms is estimated at approximately 10^13^–10^14^, and thus, outnumber the somatic cells of the host by over 10 times. Therefore, intestinal microbiota and the relative microbiome directly affect human health and disease, and have been considered as a new “organ” [1]. In the gut, microbes are physically separated from the epithelium by the mucus. In fact, the microbiome colonizes the outer layer of the mucus, and microorganisms use nutrients from the mucus itself. In healthy conditions, bacteria will only exceptionally cross the mucus to specifically interact with epithelial cells [2]. Overall, the microbiome is able to crosstalk with the host via several metabolic products (postbiotic), including short-chain fatty acids (SCFAs) like propionate, butyrate, and acetate, which originate from dietary fiber degradation, vitamins, and immunomodulatory peptides. The close interaction between gut bacteria and the host generates many benefits through the control of nutrient uptake and metabolism, strengthening the gut integrity, preventing pathogen propagation, promoting immunological tolerance to antigens, and regulating host immunity [3,4,5]. GI microbes produce several bioactive compounds which can influence the physiology of the host [6,7]; some, like vitamins, are beneficial [8], whilst others are toxic [9]. The Human Microbiome Project and MetaHit have led to an improved overview of the human-associated microbial repertoire [10,11]. The compiled data from these studies revealed that the human microbiota comprises 12 different phyla, of which 93.5% belong to Firmicutes, Bacteroidetes, Proteobacteria, and Actinobacteria. Among these, Firmicutes and Bacteroidetes dominate the gut microbiota in healthy subjects [12]. The Lachnospiraceae family is a phylogenetically and morphologically heterogeneous taxon belonging to the clostridial cluster XIVa of the phylum Firmicutes (Figure 1) [13].

Lachnospiraceae are currently described in the National Center for Biotechnology Information (NCBI) as comprising 58 genera and several unclassified strains [14]. Within Lachnospiraceae, *Blautia*, *Coprococcus*, *Dorea*, *Lachnospira*, *Oribacterium*, *Roseburia*, and L-*Ruminococcus* (*Ruminococcus* genus assigned to the Lachnospiraceae family) are the main genera that have been detected in the human intestine by metagenomics analyses. All members of Lachnospiraceae are anaerobic, fermentative, and chemoorganotrophic, and some display strong hydrolyzing activities, e.g., through the activity of pectin methyl-esterase, pectate lyase, xylanase, α-L-arabinofuranosidase, β-xylosidase α- and β-galactosidase, α- and β-glucosidase, N-acetyl-β-glucosaminidase, or α-amylase [15]. Lachnospiraceae are present in early infants, found even in the meconium [16,17,18]. However, increases in Lachnospiraceae abundances are associated with aging [19]. Lachnospiraceae abundance also increases in the intestinal lumen of subjects with different diseases, although the taxa of this family have repeatedly shown their ability to produce beneficial metabolites for the host.

The aim of this review is to unravel the physiological functions and a pathological supply of Lachnospiraceae, which are one of the core families of the human gut microbiota.

## 2. Lachnospiraceae Metabolism

**Figure 2:**
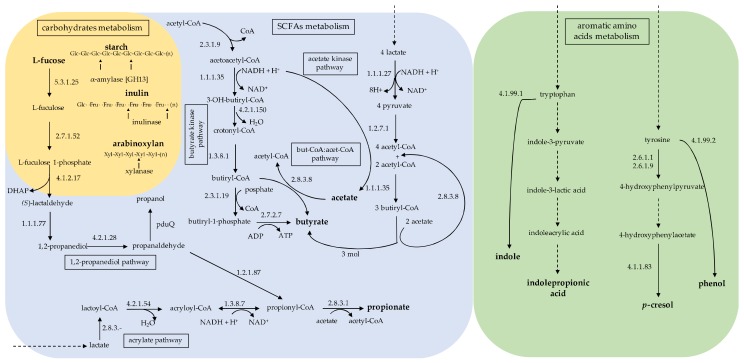
Reconstruction of the main microbial pathways associated to Lachnospiraceae in human gut. The panel in blue shows a schematic representation of the metabolic pathways involved in the biosynthesis of acetate and butyrate, as well as the main pathways of carbohydrate degradation (yellow). The green panel shows a schematic representation of metabolic pathways of aromatic amino acids involved in the biosynthesis of indole-propionic acid, indole, phenol, and *p*-cresol.

Human colonic microbiota can process a wide range of substrates, including proteins, oligopeptides, dietary polysaccharides, endogenous mucins, and glycoproteins that escape digestion by the host [20]. The metabolism of carbohydrates by the gut microbiota is a key process supplying nutrients and energy to the host. Among Firmicutes, the Lachnospiraceae, Lactobacillaceae, and Ruminococcaceae species hydrolyze starch and other sugars to produce butyrate and other SCFAs [21,22,23]. Genomic analysis of Lachnospiraceae revealed a considerable capacity to utilize diet-derived polysaccharides, including starch, inulin, and arabinoxylan, with substantial variability among species and strains (Figure 2) [24]. The growth of *Roseburia inulinivorans* on starch induces the enzymatic activity of Amy13A [25], including a GH13 amylase and two or more carbohydrate-binding modules, allowing cleavage of the α-(1,4) linkages in amylose, amylopectin, and pullulan [26]. *R. inulinivorans* can also utilize fucose through the upregulation of three fucose-inducible genes [27]. Other *Roseburia* species (i.e., *R. intestinalis*) are able to degrade xylan [28]. On the other hand, *Eubacterium eligens* and *Lachnospira pectinoschiza* were identified as pectin-utilizing Lachnospiraceae species of the human gut [29].

Prebiotics, such as fructo-oligosaccharides (FOS), inulin, lactulose, and galacto-oligosaccharidares (GOS) are non-digestible food ingredients that beneficially affect the host by selectively stimulating growth and/or the activity of one, or a limited number, of health-promoting bacteria. The increase in butyrate production was evaluated during FOS supplementation [30]. Although, the utilization of prebiotics mainly involves *Bifidobacterium*, several species of Firmicutes metabolize FOS and long-chain inulin [31]. Along this line, the butyrate-producing species *R. inulinivorans* includes strains able to grow on inulin and FOS in pure culture [32]. Within the Lachnospiraceae family, cellulolytic activity has only been assessed in the acetogenic bacterium *Bryantella formatexigens* [33].

The net contribution of SCFA to the circulating human metabolome is limited. However, these molecules play a key role in the metabolic interaction between the host and microbes (Table 1). The major products of microbial fermentation within the human colon are acetate, propionate, and butyrate, with ratios ranging from 60:20:20 to 77:15:8 [34,35,36]. Butyrate is the main SCFA produced by the *Roseburia*/*Eubacterium rectale* group, especially at a mildly acidic pH, along with the consumption of acetate [37], while other Lachnospiraceae species and strains produce formate and lactate or H_2_ in addition to butyrate [38,39]. Two different pathways are known to form butyrate from butyryl-CoA, which proceeds via either butyrate kinase or butyryl-CoA:acetate CoA-transferase [39]. *Roseburia* species and *E. rectale* share the butyryl-CoA:acetate CoA-transferase route and the same gene organization to form butyryl-CoA from two molecules of acetyl-CoA [40]. The presence of the butyryl-CoA:acetate CoA-transferase gene was also assessed in *Anaerostipes hadrus*, *Coprococcus catus*, and *Eubacterium hallii* [32,41]. On the other hand, two species of *Coprococcus* (*C. eutactus* and *C. comes*) use butyrate kinase rather than CoA-transferase for butyrate production [42].

Bacterial cross-feeding has a great impact on the balance of SCFA production and affects the efficient exploitation of substrates. The cooperation between *Roseburia intestinalis* and acetogenic species leads to butyric metabolism without the production of H_2_ [43]. *A. hadrus* and *E. hallii* can use both the isomeric forms of lactate and acetate to produce butyrate, with a net consumption of 4 mol lactate and 2 mol acetate to produce 3 mol butyrate [44,45]. On the other hand, a net production of acetate has been assessed in some *Coprococcus* species through the acetate kinase pathway [46]. The trophic interaction between *E. hallii* and the infant bifidobacterial group (*Bifidobacteirum longum* subsp. *infantis*, *B. breve*, and a strain of *B. longum* subsp. *suis*) during the degradation of L-fucose and fucosyllactoses indicated that *E. hallii* acts as a metabolically versatile species able to use intermediates of bifidobacterial oligosaccharide fermentation [47].

The production of propionate from sugar fermentation in the human gut is mainly carried out through succinate and propanediol pathways. The latter occurs in commensal bacteria *R. inulinivorans* and *Blautia* species, leading to the production of propionate and propanol from deoxy sugars fucose and rhamnose [27,41]. Moreover, *R. inulinivorans* was able to convert the propane-1,2-diol intermediate into propionate and propanol via the toxic propionaldehyde intermediate [38]. The acrylate pathway has also been shown to operate in a species of Lachnospiraceae. *Coprococcus catus* and *R. inulinivorans* are also able to switch from butyrate to propionate production via different substrates [41]. Notably, *E. hallii* is capable of metabolizing glycerol to produce 3-hydroxypropionaldehyde (3-HPA, reuterin) with reported antimicrobial activity [48]. The key enzyme that catalyzes 3-HPA production is the glycerol/diol dehydratase PduCDE, a cobalamin-dependent enzyme [49]. The conversion of glycerol to 3-HPA implies the production of cobalamin and the use of propane-1,2-diol to form propionate [50]. Mutualistic bidirectional syntropy was observed between *E. hallii* and *Akkermansia muciniphila* where, in spite of mucosa degradation, the production of vitamin B12, 1,2-propanediol, propionate, and butyrate was recorded [51]. Mucin degradation has been also assessed in some species of *Ruminococcus* and *Dorea* strains (Table 1) [52,53].

The metabolism of aromatic amino acids gives rise to uremic toxins, i.e., indoxyl sulfate (IS), p-Cresyl sulfate (*p*CS), and phenylacetylglutamine [54]. By evaluating the production of *p*-cresol and phenol in a screening study, 55 out of 153 cultured strains showed a higher concentration of *p*-cresol than the background level. In particular, a phylogenetic analysis based on the 16S rRNA gene sequences revealed that *Blautia hydrogenotrophica* YIT 10080T is one of the four strains that produced a major amount of *p*-cresol (≥100 μM) [55]. In addition, in end-stage renal disease (ESRD) patients, seven days of vancomycin administration resulted in a significant decrease in fecal *Blautia*, IS, and *p*CS levels in the serum, followed by their rebound to baseline values after the suspension of treatment [56]. IS and *p*CS are the products of tryptophan and tyrosine metabolism by the gut bacteria, and their increase following vancomycin therapy in ESRD patients indicates the resilience of the taxa generating these toxins [57]. Within a cohort of 1018 middle-aged women from TwinsUK, *Blautia* was the most common taxon associated with lower levels of indole-propionic acid (IPA), whereas a positive correlation was observed between IPA and *Coprococcus* [58]. IPA is a deamination product of tryptophan metabolism that has an important effect on host gut barrier function and antioxidant activity [20].

Flavonoids undergo various chemical modifications via hydrolysis, reduction, and other less clearly defined reactions of human gut microbiota metabolism. *Eubacterium limosum* and *Blautia* sp. MRG-PMF1—appear to metabolize flavonoids with a methoxy group, such as isoxanthohumol and icaritin, respectively [59]. An in vitro study showed that *Blautia* sp. MRG-PMF1 bio-transformed polymethoxyflavones (PMFs) in chrysin, apigenin, galangin, kaempferol, luteolin, and quercetin [60]. This class of flavonoids is involved in many biological functions, among which it exerts important role in anticancer, anti-inflammation, antiallergic, antimutagenicity, and neuroprotection activities.

## 3. Lachnospiraceae in Health

The gut microbiota is able to influence human health through the production of small molecules that accumulate in the colon and circulate systemically [20]. In the intestinal environment, some bacterial taxa degrade cellulose and hemicellulose components of indigestible plant material, and this step increases their bioavailability for host absorption. A high percentage of butyryl-CoA:acetate CoA-transferase was found in ten healthy volunteers, resulting from the presence of *E. hallii* and *E. rectale*, which were among the 10 most abundant species [32,92,93]. Complementary studies showed that *Blautia* and *Roseburia* species, often associated with a healthy state, are some of the main SCFA producers [94,95,96,97,98]. *Blautia* and *Roseburia* represent the genera most involved in the control of gut inflammatory processes, atherosclerosis, and maturation of the immune system, demonstrating that the end products of bacterial metabolism (butyrate) mediate these effects [99,100]. SCFAs were reported to be the major source of nutrition for colonic epithelial cells [98,101], especially butyrate [102,103]. SCFA activity modulates the surrounding microbial environment and directly interacts with the host immune system [104]. In addition, SCFAs lead to improved host histone epigenetic states, a shift from glycolysis to fatty acid metabolism in colonic epithelial cells, and decreased levels of inflammatory markers [99]. In mouse studies, the levels of microbiota-derived SCFAs differed according to diet [105], with a reduction in the feces of germ-free (GF) and antibiotic-treated mice compared to the control [106]. Moreover, diminished colonic regulatory T cells (Tregs), which are essential for self-antigen tolerance and autoimmune disease prevention, can be restored with SCFA administration after vancomycin treatment [107]. Specifically, propionate and acetate promote Treg accumulation in the colon [105,106], whereas butyrate and propionate enhance Treg differentiation [104,105,106]. Butyrate can also stimulate colonic Treg differentiation, when locally administered [107], or in combination with dietary starch [105,107].

Furthermore, the Lachnospiraceae family has been associated with decreased lethality from graft-versus-host disease (GVHD) after allogenic blood/marrow transplantation in two clinical settings [108]. In particular, survival improvement was assessed in patients showing a higher amount of *Blautia* [108]. Evaluate of the expression of biomarkers for the inhibition of programmed cell death, e.g., protein 1 receptor (PD-1), programmed death ligand 1 (PD-L1), and cytotoxic T lymphocyte-associated protein 4 (CTLA-4) [109] has led to mounting evidence demonstrating how the intestinal microbiota can interact with and/or influence these proteins. By studying this relationship, the authors reported a positive correlation between PD-1 and the CTLA-4 blockade and increased levels of *Dorea formicigenerans* in humans [110].

Finally, *Blautia* showed a beneficial anti-inflammatory association with an improvement of the outcomes in other clinical settings, e.g., colorectal cancer, inflammatory pouchitis after ileal pouch–anal anastomosis, and liver cirrhosis [111,112]. Bajaj et al. [112] showed that the relative abundance of Lachnospiraceae in healthy subjects was ca. 22.4%. Moreover, a recent systematic review, characterizing the composition of the pediatric gut microbiome, fixed the relative abundance of this family at ca. 16.8% [113]. Since the aim of this review was to evaluate the changes referred to disease in comparison with healthy controls, any exclusion criteria (e.g., the sequencing methods, the selection of regions used for the taxonomic assignment, and the type of samples) were applied to assess the relative abundance in healthy subjects.

## 4. Lachnospiraceae in Disease

Different studies of GI characterization have been performed with the aim to investigate the host-disease-microbes interaction. To date, not all the published evidences are concordant in asserting an active or passive role of microbiota in pathologies onset. Whether an altered microbiota is a cause or consequence, several studies reported a positive or negative statistic correlations of Lachnospiraceae taxa with pathologic status. Based on this evidence, we have reported a list of pathologies for which the significant changes in Lachnospiraceae composition appeared to be more related than other co-factors, i.e., age, gender, genetics, geography or delivery mode. Moreover, we tried to clarify the impact of main metabolites related to Lachnospiraceae on different diseases.

### 4.1. Metabolic Diseases

Obesity and closely related metabolic disorders have become highly prevalent and are dramatically rising worldwide [114]. High-fat diets (in particular, industrially products characterized by trans-fatty acids) and refined carbohydrates are the main factors contributing to the onset of metabolic syndrome, determining central obesity and insulin resistance [115]. The components of metabolic syndrome, including arterial hypertension, insulin resistance, hypertriglyceridemia, and low serum HDL-cholesterol levels, dramatically increase the risk of developing diabetes and cardiovascular disease (CVD) [116] as well as liver steatosis [117], which has recently been renamed metabolic-associated fatty liver disease (MAFLD) [118]. Therefore, the human gut microbiota plays a significant role in metabolic syndrome etiology, interacting with the diet and host metabolism [119,120,121].

By evaluating the GI microbiota in relation to the body mass index (BMI), Ley et al. [122] found an increase in Firmicutes abundance (*p*-value = 0.002) and a corresponding decrease in Bacteroidetes (*p* < 0.001), associated with a high BMI. Additionally, weight loss gradually restored the ratio of Bacteroidetes/Firmicutes [122]. Within Firmicutes, high abundances of Lachnospiraceae were positively correlated with glucose and/or lipid metabolism, indicating metabolic disturbance [69,123,124] (Table 2). Zeng et al., administering 36 weeks of a high-fat diet to mice, found increased amounts of Firmicutes compared to mice fed with a low-fat diet, particularly Lachnospiraceae [125]. As a result of correlation analysis, Kostic et al. determined that triglycerides cluster with microbes; among these, there was a positive correlation between *Blautia* and long-chain triglycerides (*p* < 0.05 or Q < 0.05, cut-off of *p* < 0.001) [126]. They also determined that the alterations in microbiota may be related to the prediabetic stage of type 1 diabetes (T1D). Lachnospiraceae actively impaired glucose metabolism, leading to inflammation and promoting the onset of T1D [126]. According to this evidence, other metagenomics studies showed that Lachnospiraceae may also be specifically associated with type 2 diabetes (T2D) in both humans and mouse models [127,128].

A new member of the family Lachnospiraceae (*Fusimonas intestini* gen. nov. strain AJ110941P) was isolated from the feces of hyperglycemic obese mice, revealing its involvement in the development of obesity and diabetes in GF mice. Colonization by the abovementioned species, within GF mice, induced significant increases in fasting blood glucose levels, liver and mesenteric adipose tissue weights, associated with a decrease in plasma insulin levels and homeostasis model assessment-β (HOMA-β) values [128]. It was recently observed that treatment with S-allyl-cysteine sulfoxide, with hypoglycemic effects, determined a decrease of Lachnospiraceae in the microbiota of diet-induced obese mice [129].

In contrast with Ley et al. [122], using a real-time PCR analysis in overweight and obese individuals, Schwiertz et al. found a decrease in Firmicutes [130], due to different data collection or sample analysis. Moreover, some studies reported that obese individuals have higher fecal concentrations of SCFAs than normal weight controls [130,131], derived from a great fermentative activity. On the other hand, a recent metagenome-wide association study revealed a loss of several butyrate-producing bacteria in faecal samples from T2D patients [92], suggesting a potential protective role uniquely for butyrate. Under this light, the dietary carbohydrates intake seems to play a crucial role in patients with metabolic disturbances, revealing that only an adequate amount of butyrate should determine beneficial effects to the host.

It is important to remark that Lachnospiraceae (in particular *Blautia*) play a key role in the metabolism of undigested carbohydrates [24]. Despite the beneficial effects concerning SCFA production from saccharolytic metabolism [94], carbohydrate digestion by GI microbiota contributes to increasing the energy derived from the diet, and thus, affecting the above-reported fasting blood glucose levels (Table 1).

### 4.2. Liver Diseases

Several studies have shown the main role of the gut microbiota in the pathogenesis and progression of the metabolic equivalent of liver steatosis, including non-alcoholic (simple) fatty liver and evolutive forms of non-alcoholic steatohepatitis (NASH), fibrosis, cirrhosis, and even hepatocellular carcinoma [132,133,134,135,136].

The gut microbiota of patients with non-alcoholic fatty liver disease (NAFLD) was enriched with Lachnospiraceae, particularly *Blautia* and *Lachnospiraceae incertae sedis* [132] (Table 2). In the same study, Shen et al. also found that patients with NASH or significant liver fibrosis display a greater abundance of Lachnospiraceae, including *Blautia* (*p* < 0.01; false discovery rate (FDR) < 0.01) [132].

As previously described, high amounts of SCFAs do not ever determine a beneficial effect. The conflicting role of SCFAs on liver health could be intended as a consequence of modern lifestyle typically characterized by an imbalanced energy intake in terms of calories (Table 1). An altered liver functionality determined a failure in lipid metabolism, thus, implying a worsening of the liver disease and the host health [67].

Compared to healthy controls, Lachnospiraceae were significantly increased in patients with primary sclerosing cholangitis (PSC)–inflammatory bowel disease (IBD), but not among patients with IBD alone [137,138]. This dysbiosis could cause a dysregulation of mucosal immunity promoting lymphocyte activation and an increase in intestinal permeability [137].

### 4.3. Kidney Diseases

Intestinal dysbiosis also occurs in chronic kidney disease (CKD) [139,140] and might actively contribute to the progression of renal failure [141,142]. The main signature of CKD dysbiosis is increased Proteobacteria [143], although increased Lachnospiraceae is also observed [140,144] (Table 2).

Along this line, patients with minimal renal dysfunction display an increase of *Blautia* and *Roseburia* species and other unclassified Lachnospiraceae (linear mixed effects regression with *p* < 0.05 and a FDR < 5%) [145]. Similarly, in CKD rats, *Blautia* contributed to the divergence of CKD rats from sham rats (principal coordinate analysis based on unweighted UniFrac distances; linear discriminant analysis (LDA) score > 2.0 with *p* < 0.05) [57]. Among the biochemical parameters and changes in the gut microbiota of CKD rats, *Blautia* was positively correlated with increased proteinuria, independent of the creatinine clearance rates and systolic blood pressure (*p* < 0.05), showing a direct association with the disease (Pearson correlation analysis; *p* < 0.01) [57]. In the following studies on the pathological metabolome of CKD rats, *Blautia* was included among taxa that showed a positive correlation with trimethylamine-N-oxide (TMAO), propanal, spermine, spermidine, N1-acetylspermidine, glycine, cinnamoylglycine, phenylacetylglycine, phenylpropionylglycine, and putrescine [57]. TMAO is the final product of the intestinal microbial metabolism of dietary lecithin, L-carnitine, and choline, and contributes to the development of atherosclerotic plaques interacting with macrophages and foam cells [146]. All these findings have demonstrated how the gut microbiota seems to have a substantial influence on systemic cardiometabolic regulation, inflammatory activation, and CVD onset by modulating the levels of bioactive metabolites [147]. Notably, plasma TMAO levels decline following the suppression of intestinal microorganisms with oral broad-spectrum antibiotics, while they nearly return to prior levels after antibiotic retraction [148]. TMAO, *p*CS, and IS are all uremic toxins or their precursors, and their accumulation results in an increased risk of CKD progression [149].

Diabetes is considered the major etiological cause of CKD onset [150], affecting kidney failure progression and cardiovascular comorbidity. There is a close relationship between Lachnospiraceae and impaired glucose metabolism. Additionally, a vegan low-protein diet (daily intake of 0.7 g/kg, as characterized by plant-based proteins and an integration between cereal and legumes to provide essential amino acids) is the main conservative therapy used to prevent the progression of kidney failure to ESRD [151]. Therefore, all these factors may contribute to the detected Lachnospiraceae overgrowth; however, further studies are needed to completely understand if and how specific operational taxonomic units (OTUs) of Lachnospiraceae are directly implicated in CKD dysbiosis.

### 4.4. Inflammatory Bowel Disease

Studies link IBD and other chronic GI illnesses to host–microbe pathways [152,153]. Children and adolescents with newly diagnosed Crohn’s disease (CD) displayed a loss in taxa belonging to the order of Clostridiales, including *Dorea*, *Blautia*, and L-*Ruminococcus* [154]. Compared to healthy controls (HC), the ileal-mucosa samples from sufferers of ileal Crohn’s disease (ICD) had significantly lower levels of L-*Ruminococcus*, *Roseburia*, *Coprococcus*, and other unclassified Clostridiales (ICD: 3.1%, HC: 15.5%; *P* = 0.017) [155]. Lower amounts of Lachnospiraceae were also previously reported in ulcerative colitis (UC) patients compared to HC (*p* < 0.001; two-tailed Student’s *t*-test) [156]. A positive correlation between Lachnospiraceae and SCFA levels was observed in UC fecal samples (R^2^ = 0.48) [157]; otherwise, it was shown that Lachnospiraceae were not affected by the UC. For these reasons, the authors concluded that the decreased abundance of Lachnospiraceae and the resulting low butyrogenesis may play a role in triggering the recurrence of UC.

The disruption of the mucus layer might promote bacterial translocation and has been associated with IBD and CD [158,159]. The mechanisms for deconstructing mucin glycan structures rely on the cooperative action of several proteases, sulfatases, and glycosidases encoded by mucin-degrading bacteria (Table 1). Most bacteria are supplied with incomplete enzyme packages specific for host mucin degradation that is likely to be achieved by a consortium of bacteria [53]. *Ruminococcus gnavus* has been identified as the major mucolytic bacteria in CD [80]. A comparative genomics analyses highlighted the presence of strain-dependent glycoside hydrolases (GHs), which is responsible for the breakdown and utilization of mucin-derived glycans [52]. With respect to UC, an increased bacterial sulfatase activity allowed *R. torques* mucolytic activity [80].

Moreover, Toll-like receptor 5 (TLR5)-deficient mice genetically sensitive to induced adherent-invasive *Escherichia coli* (AIEC) infections developed intestinal inflammation associated with microbiota alterations, among which, increases in Lachnospiraceae were observed [160]. Interestingly, members of Lachnospiraceae sampled by CD patients were previously identified as a microbial source of flagellins [161]. Hence, Jellbauer and Raffatellu supposed that the pathobiont-like AIEC triggers of the inflammation could be treated, but the increase of Firmicutes (i.e., Lachnospiraceae) remains the microbial hallmark of the depleted AIEC- infection [160].

### 4.5. Intestinal Dysbiosis Associated with the Gut–Brain Axis

Mounting evidence suggests that dysbiosis might also be involved in depression-like behavior [162,163,164]. Studies have focused on the gut–brain axis by evaluating the interactions between the GI microbiome and extraintestinal diseases. Pathways might involve reciprocal influences, linked by the sympathetic and parasympathetic system, circulating hormones, and neuropeptides [165,166,167,168]. Additionally, the vagus nerve determines the interaction between the brain and the stomach, suggesting that hormonal, neuronal, and bacterial changes in the bowel can be promptly transmitted to the brain via the vagus nerve [169].

Depression, intestinal inflammation, and changes in the gut barrier, were associated with the gut microbiome [170]. The data point to a positive correlation (Spearman’s rank correlation analysis; *p* < 0.05) between different taxa of Lachnospiraceae (specifically *Anaerostipes*, *Blautia*, *Dorea*, and *Lachnospiraceae incertae sedis*) and major depressive disorder (MDD) [164,171,172].

The gut microbiome might influence multiple sclerosis syndrome (MSS) disease [173,174], and pathways might involve the immune system [175]. Chen and co-workers compared the intestinal microbiota of MSS patients in remission with the microbiota of healthy controls. The study aimed to evaluate the active role of the microbiome in predisposition/modification of the disease. MSS patients had increased amounts of *Blautia* and *Dorea* (*P* for Wilcoxon rank-sum test < 4.38 × 10^−4^ and < 2.05 × 10^−5^, respectively) [176]. Some studies have shown that certain species of *Dorea* might promote the inflammation by supporting IFNγ production, metabolizing sialic acids, and degrading mucin [52,177]. Recently Shahi et al. [178] hypothesized that *Dorea* might play either pro or anti-inflammatory roles in MSS, depending on surrounding gut bacteria and/or cross-feeding interaction. According to the authors, in MSS patients, the growth of *Blautia* might be promoted through the utilization of gases produced by *Dorea*. The increase of *A. muciniphila*, another mucin-degrading bacterium, has been reported among MSS patients [179,180]. *Dorea* spp. and *A. muciniphila* can utilize a common pathway for mucin degradation, to induce proinflammatory responses, resulting in predisposition/ chronic inflammation. Therefore, the gut microbiota could be a cofactor responsible for the disease in genetically susceptible individuals. Further studies in this field are required.

## 5. Diet Modulates Lachnospiraceae Diversity

Although, the evidence suggests that the microbial composition can be clustered into enterotypes, the diet primarily modulates the gut microbiota composition [181,182,183,184,185]. In order to define the optimal diet for a healthy gut microbiota, a recent review unravelled the impact of single food components (macronutrients and micronutrients), salt, food additives, and different dietary habits on gut microbiota composition in term of richness and diversity [186]. Therefore, complex diets can provide a great range of growth-promoting and growth-inhibiting factors for specific phylotypes [187]. Human genomes are unable to encode most of the enzymatic patterns needed to metabolize dietary polysaccharides. Otherwise, bacterial genomes codify several enzymes involved in saccharolytic degradation, including complex carbohydrates. Plant-derived polysaccharides enter the human large intestine in the form of insoluble structures. The presence of undigested nutrients in the large intestine determined the symbiotic interaction between humans and their GI microbiota [188]. However, it is important to underline that the transit time of digesta through the colon strongly influences the activities of gut microbiota [189].

Carbohydrates are mainly fermented in the proximal colon. The intestinal fermentation of carbohydrates determined the production of hydrogen and lactate, both as final and partial metabolites. In fact, metabolic cross-feeding represents a central process within anaerobic microbial communities [190,191]. Overall, the primary activity of the caecum and colon microbiota is in the decomposition of undigested carbohydrates. Certain species are responsive to particular dietary switches of carbohydrates, mainly bacteria that are specialized to use resistant starch or non-starch polysaccharides (NSP). Some members of the *Roseburia*/*Eubacterium rectale* group were the main responders to diets enriched in resistant starch [93,192]. Other Lachnospiraceae were strongly influenced by high-NSP diets [124]. Martinez et al. tested the influence of whole grains, barley, and rice administration on the gut microbiota composition. Compared to the baseline values, whole grain consumption increased the microbial diversity (alpha diversity) and abundance of Firmicutes. This change at the phylum level was primarily derived from an increased abundance of *Blautia* and *Roseburia* [193]. By including data from dietary intake and intestinal OTUs, Di Iorio found that several species of Lachnospiraceae (specifically *Blautia wexlerae*, *B. obeum*, *B. coccoides*, *B. hydrogenotrophica*, *Coprococcus eutactus*, *Lachnospira pectinoschiza*, *Pseudobutyrivibrio xylanivorans*, and *Roseburia faecis*) were positively correlated with vegetable proteins, fiber intake, and potassium (FDR < 0.05) [194]. In fact, their ability to use complex plant material and transport degradation products of various sizes and compositions was confirmed by metagenomics studies [21]. This was probably achieved through the byproducts of ATP binding cassette (ABC) transporter proteins codified by the genomes of several Lachnospiraceae species. Furthermore, it was observed that *Roseburia* and *Lachnospira* were strongly associated with vegetable diets (vegetarian and vegan diets), and also displayed a negative association (*p* < 0.01) with the omnivore diet. On the same line, a recent study positively correlated *Lachnospira* to the intake of beta-carotene, vitamin E and vegetable fat whereas a negative correlation was found with meat, total proteins, and cholesterol (FDR <0.05) [185]. Oppositely, L-*Ruminococcus*, *Blautia*, and *Lachnobacterium* were included in the cluster of bacterial taxa that positively correlated with animal-derived nutrients and negatively correlated with vegetable-based diet patterns [195].

High fat and sugar levels are the mainstay of the Western diet. As mentioned above, different OTUs of Lachnospiraceae were related to altered lipid metabolism and, thus, to obesity [69,124,126], or specific nutrients, such as saturated and total fats [195]. By analyzing the chemical composition at a single-cell level in C57BL/6NCrl mice, studies found that the microbiota composition, particularly amounts of Lachnospiraceae, was altered by high-fat feeding [196]. This microbial imbalance may originate from phylotype dynamic shifts, but also from altered Lachnospiraceae metabolic activity [196]. It is important to empathize that some dietary fats, particularly omega-3 polyunsaturated fatty acids (omega-3-PUFA), may improve human health reducing the risk of the coronary heart disease death and the develop of breast cancer [197,198,199]. Some Lachnospiraceae taxa showed that two weeks of diet implemented with omega-3-PUFA determined an increase of their abundances [200]. Specifically, at genus level *Blautia* and *Coprococcus* significantly increased, while at the species level, *Roseburia* spp./*Eubacterium rectale* became the predominant species [200]. Menni et al. [201] found a positive association between 36 OTUs and the serum levels of docosahexaenoic acid (DHA); 21 out of 36 OTUs belong to the Lachnospiraceae. DHA is one of the main structural lipids in the mammalian brain [202], positively linked to the prevention of numerous human pathologies including cancer and heart disease [203].

The involvement of Lachnospiraceae species in protein metabolism is less clear. In a previous trial, species of this family showed a marked negative correlation with the protein intake, especially animal proteins (FDR < 0.05) [194]. Additionally, in a study performed using a murine model, the relative abundance of Lachnospiraceae decreased after the consumption of a high-protein/low-carbohydrate diet, compared to a normal diet [204]. Although Lachnospiraceae appear to be less involved in proteolytic metabolism, the evidence provided could be the starting point for specific studies to link Lachnospiraceae to dietary digestion.

## 6. Conclusions

The evidence from different studies shows that Lachnospiraceae might influence healthy functions, although different genera and species of this family are increased in diseases. To the best of our knowledge, metabolic syndrome, obesity, diabetes, liver diseases, IBD, and CKD are all inflammatory conditions involving the Lachnospiraceae family or specific taxa of Lachnospiraceae. Furthermore, they appear to be involved in depressive syndromes and multiple sclerosis syndrome.

A deeper understanding of the mechanisms involved in interactions with the host will represent the main future challenge, with a specific focus on the immunological details and especially the diet interactions stimulating or restricting the presence of microbial pathways or the production of specific metabolites. The ultimate aim is to improve intestinal epithelial integrity and health. Further studies are needed to understand the potential impact of microbial-targeted therapies, including the modulation of Lachnospiraceae, with the end goal of their utilization in the prevention and treatment of both intestinal and extraintestinal diseases.

## Figures and Tables

**Table 1:** Summary of the main metabolic pathways and corresponding Lachnospiraceae taxa involved in the production of compounds affecting human health. The beneficial and harmful effects are referred to the main diseases showing Lachnospiraceae variations.

Taxa	Pathways (EC)	Metabolites	Beneficial Effect *		Harmful Effect	
	Butyril-CoA:acetate CoA trasferase (2.8.3.8)	butyrate	MD LD IBD	Strengthen the intestinal barrier through up-regulation of tight junctions and mucin production by enterocytes [61].	MD LD	
				Anti-inflammatory effects by induction of regulatory T cells, downregulation of pro-inflammatory cytokines and the Toll-like receptor (TLR) 4 receptors [62].		
				Activation of G protein-coupled receptor (GPR) 43 involved in the modulation of inflammation and stimulation of glucagon-like peptides (GLP) 1 and gastric inhibitory polypeptide; modulate appetite, reinforce insulin sensitivity and glucose metabolism [63,64].		
*Eubacterium rectale*						
*Roseburia* spp.,						
*E. halii* L2-7,						
*Anaerostipes hadrus* SSC/2, *Coprococcus catus* GD/7,				Activation of fatty acid oxidation and de novo synthesis and lipolysis inhibition, which in turn, decrease circulating lipid plasma levels and body weight [65].		
*Blautia* spp.						

				GPR 43 binding suppresses colon inflammation therefore protect liver and down- regulate insulin signal transduction in adipose tissue [66].		Elevated energy extraction in form of SCFAs related to a high intake of dietary carbohydrates [67].
			MD LD	Lower expression of peroxisome proliferator-activated receptor-γ, and stimulation of uncoupling protein 2 and stimulate oxidative metabolism in liver and adipose tissue [70].		Intestinotrophic effects of SCFAs mediated by GLP-2 which contributes to the development or maintenance of obesity through elevated intestinal absorption of energy (kcal) intake [68].
			MD	Inhibition of Histone Deacetylases by altering the acetylation pattern of H3 and H4 histones and inducing beta-cell proliferation by inhibiting the p38/ERK apoptotic pathway [71,72].		Dyslipidemia due to elevation of cholesterol and triglycerides that increasing the levels of Acetyl-CoA in obese patient and metabolic disturbance [69].
*C. comes ATCC 27758*,	Butyrate kinase		MDDMSS			
*C. eutactus L2-50*	(2.7.2.7)		IBD	Significantly reduced circulating LPS levels [73]. Activation of GPR109A and inhibition of AKT and nuclear factor-κB p65 signaling pathways in IBD in mice [74].		
			CKD			
			MSS	Increase anti-inflammatory CD4+ regulatory T cells and decrease pro-inflammatory Th1 and Th17 cells of in central nervous system. [75]. Upregulate tight junction and proteins claudin-5 and restore the blood-brain barrier permeability [76].		
*Ruminococcus inulinivorans* A2-194,	Propanediol pathways (4.2.1.28, 1.2.1.87, 2.8.3.1.)	propionate	MD LD CKD	SCFA-stimulated GPR41 induce leptin production by adipocytes and lipid profile regulation [63,64]. Reduction of visceral fat and liver fat [77].	MD	Substantial amounts of propionate entering into the mitochondrial tricarboxylic acid (TCA) cycle bypass the first four TCA enzymes, causing a shift in the cycle with a potential toxic effect [78,79].
*R. gnavus* ATCC 29149,						
*R. torques* L2-14, *Blautia obeum* A2.162,						
*E. hallii*						
*C. catus*,	Acrylate pathway (4.2.1.4, 1.3.8.7, 2.8.3.1.)					
*Clostridium* sp. MSTE9 (cluster XIVb)						
*R. gnavus* ATCC 29149,	Mucin degradation (glycoside hydrolases (GH))				IBD	Disproportionate increase of mucolytic bacteria could explain increased total mucosa-associated bacteria in IBD [80].
*R. gnavus* ATCC 35913,						
*R. torques*,						
*Dorea formicigenerans*,						
*D. longicatena*						
*Roseburia intestinalis* L1-82,		acetate	MD LD CKD IBD	Inhibition of entero-pathogens; reduction of luminal pH, and increases the absorption of dietary nutrient [81,82]. Trophic effect on the colonic epithelium by raising the mucosal blood flux [83].	MD	Increased production leads to activation of the parasympathetic nervous system and stimulation of insulin secretion. The role of acetate in driving obesity depends on the gut microbiota and on dietary fiber intake [84]. Transported to the portal circulation across the colonic mucosa, acetate passes through the liver and is regained in peripheral blood, where it is adsorbed by tissues involved in the rise of cholesterol synthesis [85].
*R. intestinalis* L1-952,						
*R. intestinalis* L1-8152,	Acetate kinase					
*Coprococcus catus*,	(2.7.2.1)		LD	De novo lipogenesis and cholesterol genesis in the liver [86].		
*Blautia sp. YL58*,				Marked reduction in lipid accumulation in the adipose tissue, protects against accumulation of fat in the liver, improving the glucose tolerance [87].		
*B. obeum*,						
*B. hansenii*						
*Blautia hydrogenotrophica* YIT 10080T,		*p*-cresol			CKD	The derived serum p-Cresyl sulphate a protein-derived uremic toxin is linked to cardiovascular and kidney damage [20].
	Tyrosine					
*B. obeum*.	(2.6.1.1, 2.6.1.9, 4.1.1.83)					
*Clostridium saccharolyticum* WM1	Tyrosine (4.1.99.2)	phenol				
*Oribacterium sinus*,	Tryptophan (4.1.99.1)	indole	MD LD CKD IBD	Activation of aryl-hydrocarbon receptor by microbially derived indoles, these molecules promotes tissue repair and homeostasis involving interleukin (IL)-22 [88].	CKD	Indole and indoxyl sulfate affect arterial blood pressure via peripheral and central mechanisms dependent on serotonin signaling and contribute do cardiovascular disease in renal insufficiency [89].
*Lachnospiraceae*						
*Coprococcus*	Tryptophan	indole-propionic acid	MD	Engage the pregnane X receptor, leading to the upregulation of genes that regulate intestinal permeability and to the downregulation of TNF-α expression by enterocytes [90].		
			MSS	Potent radical scavenging activity and neuroprotective properties [91].		

* The beneficial/harmful effects are referred to the relative diseases showed on the left side; **Abbreviations:** **MD**, metabolic diseases; **LD**, liver disease; **IBD**, inflammatory bowel disease; **MDD**, major depressive disorder; **MSS**, multiple sclerosis syndrome; **CKD**, chronic kidney disease; **LPS**, lipopolysaccharide; **NF-κB**, nuclear factor-κB; **TNF-α**, tumor necrosis factor.

**Table 2:** Taxa of Lachnospiraceae detected in different diseases in humans and animal models. Number of samples and changes in taxon are also indicated.

Taxon	Change	Principal Disease	Patient Type/Model (*Number*)	Ref.
*Lachnospira* and *Coprococcus*	↑	MD	Women with obesity + metabolic syndrome (*25*)	[69]
Lachnospiraceae	↑	MD	Individuals with glucose metabolism disorder (*20*)	[123]
Lachnospiraceae	↑	MD	Male patients (*14*)	[124]
Lachnospiraceae	↑	MD	Male C57BL/6 mice (*12*)	[125]
*Blautia*	↑	Prediabetic stage	Infants with serum autoantibody positivity (*11*)	[126]
*Blautia*	↑	Diabetes T1	Infants with T1D (*4*)	[126]
Lachnospiraceae	↑	Diabetes T2	Patients with T2D (*71*)	[127]
Lachnospiraceae	↑	Diabetes T2	Cg-Dock7^m^ +/+Lepr^db^/J [*db/db*] mice (*4*)	[128]
*Blautia* and *Lachnospiraceae incertae sedis*	↑	NAFLD	Male patients (*19*)	[132]
*Blautia*	↑	NASH	Male patients (*4*)	[132]
Lachnospiraceae	↑	PSC–IBD	Patients (*11*)	[137]
*Blautia*	↑	PSC	Patients (*20*), 19 of which had concomitant IBD	[138]
Lachnospiraceae	↑	IgAN	Patients IgAN progressor (*16*) and patients IgAN non-progressors (*16*)	[140]
Lachnospiraceae	↑	CKD	Male Sprague–Dawley rats (*6*)	[144]
*Blautia* and *Roseburia*	↑	Renal dysfunction	Individuals with eGFR < 60mL/min/1.73m^2^ (*62*)	[145]
*Blautia*	↑	CKD	Nephrectomy rats (*6*)	[57]
Clostridiales (*Dorea*, *Blautia*, L-*Ruminococcus*)	↓	CD	Children and adolescents (<17 years) with newly diagnosed CD (*447*)	[154]
L-*Ruminococcus*, *Roseburia*, *Coprococcus*	↓	ICD	Patients with ICD (*7*)	[155]
L-*Ruminococcus*, *Roseburia*, *Coprococcus*	↓	CCD	Patients with CCD and with normal ileum (*6*)	[155]
Lachnospiraceae	↓	CD	Tissue samples from CD patients (*68*)	[156]
Lachnospiraceae	↓	UC	Tissue samples from UC patients (*61*)	[156]
Lachnospiraceae	↑	AIEC infections	TLR5-deficient mice (*n. of samples not shown*)	[160]
Lachnospiraceae	↑	CD	Bacterial isolation from mouse cecum (*1*)	[161]
*Anaerostipes*, *Blautia*, *Dorea*, and *Lachnospiraceae incertae sedis*	↑	MDD	MDD subjects (*39*) were drug naive and MDD subjects (*19*) treated with various anti-depressants	[164]
*Blautia* and *Lachnospiraceae incertae sedis*	↑	MDD	Active-MDD patients (*29*) and responding-MDD patients (*17*)	[172]
*Blautia* and *Dorea*	↑	MSS	Patients (*31*)	[176]

**Abbreviations:↑**, increase; **↓**, decrease; **MD**, metabolic diseases; **NAFLD**, non-alcoholic fatty liver disease; **NASH**, non-alcoholic steatohepatitis; **PSC**, primary sclerosing cholangitis; **IBD**, inflammatory bowel disease; **IgAN**, immunoglobulin A nephropathy; **CKD**, chronic kidney disease; **AIEC**, adherent-invasive *Escherichia coli*; **CD**, Crohn’s disease; **ICD**, ileal Crohn’s disease; **CCD**, Crohn’s disease restricted to the colon; **UC**, ulcerative colitis; **MDD**, major depressive disorder; **MSS**, multiple sclerosis. syndrome.

## References

[1] Baquero F., Nombela C. (2012). The microbiome as a human organ. Clin. Microbiol. Infect..

[2] Atarashi K., Tanoue T., Ando M., Kamada N., Nagano Y., Narushima S., Suda W., Imaoka A., Setoyama H., Nagamori T. (2015). Th17 Cell Induction by Adhesion of Microbes to Intestinal Epithelial Cells. Cell.

[3] Gensollen T., Iyer S.S., Kasper D.L., Blumberg R.S. (2016). How colonization by microbiota in early life shapes the immune system. Science.

[4] Power S.E., O’Toole P.W., Stanton C., Ross R.P., Fitzgerald G.F. (2014). Intestinal microbiota, diet and health. Br. J. Nutr..

[5] Valdes A.M., Walter J., Segal E., Spector T.D. (2018). Role of the gut microbiota in nutrition and health. BMJ.

[6] Gentile C.L., Weir T.L. (2018). The gut microbiota at the intersection of diet and human health. Science.

[7] Zhang N., Ju Z., Zuo T. (2018). Time for food: The impact of diet on gut microbiota and human health. Nutrition.

[8] Crittenden R.G., Martinez N.R., Playne M.J. (2003). Synthesis and utilisation of folate by yoghurt starter cultures and probiotic bacteria. Int. J. Food Microbiol..

[9] Riwes M., Reddy P. (2018). Microbial metabolites and graft versus host disease. Am. J. Transplant..

[10] Huttenhower C., Gevers D., Knight R., Abubucker S., Badger J.H., Chinwalla A.T., Creasy H.H., Earl A.M., FitzGerald M.G., Fulton R.S. (2012). Structure, function and diversity of the healthy human microbiome. Nature.

[11] Li J., Jia H., Cai X., Zhong H., Feng Q., Sunagawa S., Arumugam M., Kultima J.R., Prifti E., Nielsen T. (2014). An integrated catalog of reference genes in the human gut microbiome. Nat. Biotechnol..

[12] Lozupone C.A., Stombaugh J.I., Gordon J.I., Jansson J.K., Knight R. (2012). Diversity, stability and resilience of the human gut microbiota. Nature.

[13] Rainey F.A., Family V., De Vos P., Garrity G.M., Jones D., Krieg N.R., Ludwig W., Rainey F.A., Schleifer K.H., Whitman W.B. (2009). Lachnospiraceae fam. nov. Bergey’s Manual of Systematic Bacteriology.

[14] Sayers E.W., Barrett T., Benson D.A., Bolton E., Bryant S.H., Canese K., Chetvernin V., Church D.M., DiCuccio M., Federhen S. (2009). Database resources of the national center for biotechnology information. Nucleic Acids Res..

[15] Stackebrandt E., Rosenberg E., DeLong E.F., Lory S., Stackebrandt E., Thompson F. (2014). The Family Lachnospiraceae. The Prokaryotes.

[16] Ding Y., Xiao L., Guo J., Jiong L.U., Hao X.U., Hou M., Ben X. (2017). Intestinal microbiota in neonates within three days after birth. Chin. J. Perinat. Med..

[17] Sagheddu V., Patrone V., Miragoli F., Puglisi E., Morelli L. (2016). Infant early gut colonization by Lachnospiraceae: High frequency of Ruminococcus gnavus. Front. Pediatr.

[18] Sohn K., Underwood M.A. (2017). Prenatal and postnatal administration of prebiotics and probiotics. Semin Fetal Neonatal Med..

[19] Odamaki T., Kato K., Sugahara H., Hashikura N., Takahashi S., Xiao J.Z., Abe F., Osawa R. (2016). Age-related changes in gut microbiota composition from newborn to centenarian: A cross-sectional study. BMC Microbiol..

[20] Van Treuren W., Dodd D. (2020). Microbial contribution to the human metabolome: Implications for health and disease. Annu. Rev. Pathol. Mech. Dis..

[21] Biddle A., Stewart L., Blanchard J., Leschine S. (2013). Untangling the genetic basis of fibrolytic specialization by Lachnospiraceae and Ruminococcaceae in diverse gut communities. Diversity.

[22] Devillard E., McIntosh F.M., Duncan S.H., Wallace R.J. (2007). Metabolism of linoleic acid by human gut bacteria: Different routes for biosynthesis of conjugated linoleic acid. J. Bacteriol..

[23] Wong J., Piceno Y.M., DeSantis T.Z., Pahl M., Andersen G.L., Vaziri N.D. (2014). Expansion of urease-and uricase-containing, indole-and p-cresol-forming and contraction of short-chain fatty acid-producing intestinal microbiota in ESRD. Am. J. Nephrol..

[24] Sheridan P.O., Martin J.C., Lawley T.D., Browne H.P., Harris H.M., Bernalier-Donadille A., Duncan S.H., O’Toole P.W., Scott K.P., Flint H.J. (2016). Polysaccharide utilization loci and nutritional specialization in a dominant group of butyrate-producing human colonic Firmicutes. Microb. Genom..

[25] Scott K.P., Martin J.C., Chassard C., Clerget M., Potrykus J., Campbell G., Mayer C.D., Young P., Rucklidge G., Ramsay A.G. (2011). Substrate-driven gene expression in Roseburia inulinivorans: Importance of inducible enzymes in the utilization of inulin and starch. Proc. Natl. Acad. Sci. USA.

[26] Ramsay A.G., Scott K.P., Martin J.C., Rincon M.T., Flint H.J. (2006). Cell-associated α-amylases of butyrate-producing Firmicute bacteria from the human colon. Microbiology.

[27] Scott K.P., Martin J.C., Campbell G., Mayer C.D., Flint H.J. (2006). Whole-genome transcription profiling reveals genes up-regulated by growth on fucose in the human gut bacterium “Roseburia inulinivorans”. J. Bacteriol..

[28] Chassard C., Goumy V., Leclerc M., Del’homme C., Bernalier-Donadille A. (2007). Characterization of the xylan-degrading microbial community from human faeces. FEMS Microbiol. Ecol..

[29] Flint H.J., Scott K.P., Duncan S.H., Louis P., Forano E. (2012). Microbial degradation of complex carbohydrates in the gut. Gut Microbes.

[30] Duncan S.H., Scott K.P., Ramsay A.G., Harmsen H.J., Welling G.W., Stewart C.S., Flint H.J. (2003). Effects of alternative dietary substrates on competition between human colonic bacteria in an anaerobic fermentor system. Appl. Environ. Microbiol..

[31] Rossi M., Corradini C., Amaretti A., Nicolini M., Pompei A., Zanoni S., Matteuzzi D. (2005). Fermentation of fructooligosaccharides and inulin by bifidobacteria: A comparative study of pure and fecal cultures. Appl. Environ. Microbiol..

[32] Louis P., Young P., Holtrop G., Flint H.J. (2010). Diversity of human colonic butyrate-producing bacteria revealed by analysis of the butyryl-CoA: Acetate CoA-transferase gene. Environ. Microbiol..

[33] Wolin M.J., Miller T.L., Collins M.D., Lawson P.A. (2003). Formate-Dependent Growth and Homoacetogenic Fermentation by a Bacterium from Human Feces: Description of Bryantella formatexigens gen. nov., sp. nov. Appl. Environ. Microbiol..

[34] Cummings J., Pomare E.W., Branch W.J., Naylor C.P., Macfarlane G.T. (1987). Short chain fatty acids in human large intestine, portal, hepatic and venous blood. Gut.

[35] Den Besten G., Lange K., Havinga R., van Dijk T.H., Gerding A., van Eunen K., Reijngoud D.J. (2013). Gut-derived short-chain fatty acids are vividly assimilated into host carbohydrates and lipids. Am. J. Physiol..

[36] Macfarlane G.T., Gibson G.R., Cummings J.H. (1992). Comparison of fermentation reactions in different regions of the human colon. J. Appl. Bacteriol..

[37] Kettle H., Louis P., Holtrop G., Duncan S.H., Flint H.J. (2015). Modelling the emergent dynamics and major metabolites of the human colonic microbiota. Environ. Microbiol..

[38] Tamanai-Shacoori Z., Smida I., Bousarghin L., Loreal O., Meuric V., Fong S.B., Bonnaure-Mallet M., Jolivet-Gougeon A. (2017). Roseburia spp.: A marker of health?. Future Microbiol..

[39] Louis P., Flint H.J. (2009). Diversity, metabolism and microbial ecology of butyrate-producing bacteria from the human large intestine. FEMS Microbiol. Lett..

[40] Louis P., Flint H.J. (2017). Formation of propionate and butyrate by the human colonic microbiota. Environ. Microbiol..

[41] Reichardt N., Duncan S.H., Young P., Belenguer A., Leitch C.M., Scott K.P., Flint H.J., Louis P. (2014). Phylogenetic distribution of three pathways for propionate production within the human gut microbiota. ISME J..

[42] Louis P., Duncan S.H., McCrae S.I., Millar J., Jackson M.S., Flint H.J. (2004). Restricted distribution of the butyrate kinase pathway among butyrate-producing bacteria from the human colon. J. Bacteriol..

[43] Chassard C., Bernalier-Donadille A. (2006). H2 and acetate transfers during xylan fermentation between a butyrate-producing xylanolytic species and hydrogenotrophic microorganisms from the human gut. FEMS Microbiol. Lett..

[44] Allen-Vercoe E., Daigneault M., White A., Panaccione R., Duncan S.H., Flint H.J., O’Neal L., Lawson P.A. (2012). Anaerostipes hadrus comb. nov., a dominant species within the human colonic microbiota; reclassification of Eubacterium hadrum Moore et al. 1976. Anaerobe.

[45] Duncan S.H., Louis P., Flint H.J. (2004). Lactate-utilizing bacteria, isolated from human feces, that produce butyrate as a major fermentation product. Appl. Environ. Microbiol..

[46] Duncan S.H., Barcenilla A., Stewart C.S., Pryde S.E., Flint H.J. (2002). Acetate utilization and butyryl coenzyme A (CoA): Acetate-CoA transferase in butyrate-producing bacteria from the human large intestine. Appl. Environ. Microbiol..

[47] Schwab C., Ruscheweyh H.J., Bunesova V., Pham V.T., Beerenwinkel N., Lacroix C. (2017). Trophic interactions of infant bifidobacteria and Eubacterium hallii during L-fucose and fucosyllactose degradation. Front. Microbiol..

[48] Fekry M.I., Engels C., Zhang J., Schwab C., Lacroix C., Sturla S.J., Chassard C. (2016). The strict anaerobic gut microbe Eubacterium hallii transforms the carcinogenic dietary heterocyclic amine 2-amino-1-methyl-6-phenylimidazo [4, 5-b] pyridine (PhIP). Environ. Microbiol. Rep..

[49] Morita H., Toh H., Fukuda S., Horikawa H., Oshima K., Suzuki T., Murakami M., Hisamatsu S., Kato Y., Takizawa T. (2008). Comparative genome analysis of Lactobacillus reuteri and Lactobacillus fermentum reveal a genomic island for reuterin and cobalamin production. DNA Res..

[50] Engels C., Ruscheweyh H.J., Beerenwinkel N., Lacroix C., Schwab C. (2016). The common gut microbe Eubacterium hallii also contributes to intestinal propionate formation. Front. Microbiol..

[51] Belzer C., Chia L.W., Aalvink S., Chamlagain B., Piironen V., Knol J., de Vos W.M. (2017). Microbial metabolic networks at the mucus layer lead to diet-independent butyrate and vitamin B12 production by intestinal symbionts. MBio.

[52] Crost E.H., Tailford L.E., Le Gall G., Fons M., Henrissat B., Juge N. (2013). Utilisation of mucin glycans by the human gut symbiont Ruminococcus gnavus is strain-dependent. PLoS ONE.

[53] Crost E.H., Tailford L.E., Monestier M., Swarbreck D., Henrissat B., Crossman L.C., Juge N. (2016). The mucin-degradation strategy of Ruminococcus gnavus: The importance of intramolecular trans-sialidases. Gut Microbes.

[54] Meyer T.W., Hostetter T.H. (2012). Uremic solutes from colon microbes. Kidney Int..

[55] Saito Y., Sato T., Nomoto K., Tsuji H. (2018). Identification of phenol-and p-cresol-producing intestinal bacteria by using media supplemented with tyrosine and its metabolites. FEMS Microbiol. Ecol..

[56] Nazzal L., Roberts J., Singh P., Jhawar S., Matalon A., Gao Z., Holzman R., Liebes L., Blaser M.J., Lowenstein J. (2017). Microbiome perturbation by oral vancomycin reduces plasma concentration of two gut-derived uremic solutes, indoxyl sulfate and p-cresyl sulfate, in end-stage renal disease. Nephrol. Dial. Transpl..

[57] Feng Y.L., Cao G., Chen D.Q., Vaziri N.D., Chen L., Zhang J., Wang M., Guo Y., Zhao Y.Y. (2019). Microbiome–metabolomics reveals gut microbiota associated with glycine-conjugated metabolites and polyamine metabolism in chronic kidney disease. Cell Mol. Life Sci..

[58] Menni C., Hernandez M.M., Vital M., Mohney R.P., Spector T.D., Valdes A.M. (2019). Circulating levels of the anti-oxidant indoleproprionic acid are associated with higher gut microbiome diversity. Gut Microbes.

[59] Possemiers S., Heyerick A., Robbens V., De Keukeleire D., Verstraete W. (2005). Activation of proestrogens from hops (Humulus lupulus L.) by intestinal microbiota; conversion of isoxanthohumol into 8-prenylnaringenin. J. Agric. Food Chem..

[60] Burapan S., Kim M., Han J. (2017). Demethylation of polymethoxyflavones by human gut bacterium, Blautia sp. MRG-PMF1. J. Agric. Food Chem..

[61] Brahe L.K., Astrup A., Larsen L.H. (2013). Is butyrate the link between diet, intestinal microbiota and obesity-related metabolic diseases?. Obes. Rev..

[62] Portune K.J., Benítez-Páez A., Del Pulgar E.M.G., Cerrudo V., Sanz Y. (2017). Gut microbiota, diet, and obesity-related disorders—The good, the bad, and the future challenges. Mol. Nutr. Food Res..

[63] Layden B.T., Angueira A.R., Brodsky M., Durai V., Lowe W.L. (2013). Short chain fatty acids and their receptors: New metabolic targets. Transl. Res..

[64] Puddu A., Sanguineti R., Montecucco F., Viviani G.L. (2014). Evidence for the gut microbiota short-chain fatty acids as key pathophysiological molecules improving diabetes. Mediators Inflamm..

[65] Esgalhado M., Kemp J.A., Damasceno N.R., Fouque D., Mafra D. (2017). Short-chain fatty acids: A link between prebiotics and microbiota in chronic kidney disease. Future Microbiol..

[66] Maslowski K.M., Vieira A.T., Ng A., Kranich J., Sierro F., Yu D., Schilter H.C., Rolph M.S., Mackay F., Artis D. (2009). Regulation of inflammatory responses by gut microbiota and chemoattractant receptor GPR43. Nature.

[67] Zhu L., Baker R.D., Baker S.S. (2015). Gut microbiome and nonalcoholic fatty liver diseases. Pediatric. Res..

[68] Tappenden K.A., McBurney M.I. (1998). Systemic short-chain fatty acids rapidly alter gastrointestinal structure, function, and expression of early response genes. Dig. Dis. Sci..

[69] Chávez-Carbajal A., Nirmalkar K., Pérez-Lizaur A., Hernández-Quiroz F., Ramírez-del-Alto S., García-Mena J., Hernández-Guerrero C. (2019). Gut Microbiota and Predicted Metabolic Pathways in a Sample of Mexican Women Affected by Obesity and Obesity Plus Metabolic Syndrome. Int. J. Mol. Sci..

[70] Den Besten G., Bleeker A., Gerding A., van Eunen K., Havinga R., van Dijk T.H., Oosterveer M.H., Jonker J.W., Groen A.K., Reijngoud D.J. (2015). Short-Chain Fatty Acids protect against High-Fat Diet- Induced Obesity via a PPARgamma-dependent switch from lipogenesis to fat oxidation. Diabetes.

[71] Misztak P., Pańczyszyn-Trzewik P., Sowa-Kućma M. (2018). Histone deacetylases (HDACs) as therapeutic target for depressive disorders. Pharmacol. Rep..

[72] Faraco G., Cavone L., Chiarugi A. (2011). The therapeutic potential of HDAC inhibitors in the treatment of multiple sclerosis. Mol. Med..

[73] Gonzalez A., Krieg R., Massey H.D., Carl D., Ghosh S., Gehr T.W., Ghosh S.S. (2019). Sodium butyrate ameliorates insulin resistance and renal failure in CKD rats by modulating intestinal permeability and mucin expression. Nephrol. Dial. Transpl..

[74] Chen G., Ran X., Li B., Li Y., He D., Huang B., Wang W. (2018). Sodium butyrate inhibits inflammation and maintains epithelium barrier integrity in a TNBS-induced inflammatory bowel disease mice model. EBioMedicine.

[75] Melbye P., Olsson A., Hansen T.H., Søndergaard H.B., Bang Oturai A. (2019). Short-chain fatty acids and gut microbiota in multiple sclerosis. Acta Neurol. Scand..

[76] Braniste V., Al-Asmakh M., Kowal C., Anuar F., Abbaspour A., Tóth M., Korecka A., Bakocevic N., Guan N.L., Kundu P. (2014). The gut microbiota influences blood-brain barrier permeability in mice. Sci. Transl. Med..

[77] Soty M., Penhoat A., Amigo-Correig M., Vinera J., Sardella A., Vullin-Bouilloux F., Zitoun C., Houberdon I., Mithieux G. (2015). A gut-brain neural circuit controlled by intestinal gluconeogenesis is crucial in metabolic health. Mol. Metab..

[78] Frye R.E., Rose S., Chacko J., Wynne R., Bennuri S.C., Slattery J.C., Tippett M., Delhey L., Melnyk S., Kahler S.G. (2016). Modulation of mitochondrial function by the microbiome metabolite propionic acid in autism and control cell lines. Transl. Psychiat..

[79] Sanna S., van Zuydam N.R., Mahajan A., Kurilshikov A., Vila A.V., Võsa U., Mujagic Z., Masclee A.A.M., Jonkers D.M.A.E., Oosting M. (2019). Causal relationships among the gut microbiome, short-chain fatty acids and metabolic diseases. Nat. Genet..

[80] Png C.W., Lindén S.K., Gilshenan K.S., Zoetendal E.G., McSweeney C.S., Sly L.I., McGuckin M.A., Florin T.H. (2010). Mucolytic Bacteria With Increased Prevalence in IBD Mucosa AugmentIn VitroUtilization of Mucin by Other Bacteria. Am. J. Gastroenterol..

[81] Slavin J. (2013). Fiber and prebiotics: Mechanisms and health benefits. Nutrients.

[82] Ríos-Covián D., Ruas-Madiedo P., Margolles A., Gueimonde M., de los Reyes-Gavilán C.G., Salazar N. (2016). Intestinal short chain fatty acids and their link with diet and human health. Front. Microbiol..

[83] Vernocchi P., Del Chierico F., Putignani L. (2016). Gut microbiota profiling: Metabolomics based approach to unravel compounds affecting human health. Front. Microbiol..

[84] Perry R.J., Peng L., Barry N.A., Cline G.W., Zhang D., Cardone R.L., Petersen K.F., Kibbey R.G., Goodman A.L., Shulman G.I. (2016). Acetate mediates a microbiome–brain–β-cell axis to promote metabolic syndrome. Nature.

[85] Murugesan S., Nirmalkar K., Hoyo-Vadillo C., García-Espitia M., Ramírez-Sánchez D., García-Mena J. (2018). Gut microbiome production of short-chain fatty acids and obesity in children. Eur. J. Clin. Microbiol..

[86] Morrison D.J., Preston T. (2016). Formation of short chain fatty acids by the gut microbiota and their impact on human metabolism. Gut Microbes.

[87] Yamashita H., Fujisawa K., Ito E., Idei S., Kawaguchi N., Kimoto M., Hiemori M., Tsuji H. (2007). Improvement of obesity and glucose tolerance by acetate in type 2 diabetic otsuka long-evans tokushima fatty (OLETF) rats. Biosci. Biotechnol. Biochem..

[88] Zelante T., Iannitti R.G., Cunha C., De Luca A., Giovannini G., Pieraccini G., Zecchi R., D’Angelo C., Massi-Benedetti C., Fallarino F. (2013). Tryptophan catabolites from microbiota engage aryl hydrocarbon receptor and balance mucosal reactivity via interleukin-22. Immunity.

[89] Huć T., Nowinski A., Drapala A., Konopelski P., Ufnal M. (2018). Indole and indoxyl sulfate, gut bacteria metabolites of tryptophan, change arterial blood pressure via peripheral and central mechanisms in rats. Pharmacol. Res..

[90] Venkatesh M., Mukherjee S., Wang H., Li H., Sun K., Benechet A.P., Qiu Z., Maher L., Redinbo M.R., Phillips R.S. (2014). Symbiotic bacterial metabolites regulate gastrointestinal barrier function via the xenobiotic sensor PXR and Toll-like receptor 4. Immunity.

[91] Chyan Y.J., Poeggeler B., Omar R.A., Chain D.G., Frangione B., Ghiso J., Pappolla M.A. (1999). Potent neuroprotective properties against the Alzheimer β-amyloid by an endogenous melatonin-related indole structure, indole-3-propionic acid. J. Biol. Chem..

[92] Qin J., Li R., Raes J., Arumugam M., Burgdorf K.S., Manichanh C., Nielsen T., Pons N., Levenez F., Yamada T. (2010). A human gut microbial gene catalogue established by metagenomic sequencing. Nature.

[93] Walker A.W., Ince J., Duncan S.H., Webster L.M., Holtrop G., Ze X., Brown D., Stares M.D., Scott P., Bergerat A. (2011). Dominant and diet-responsive groups of bacteria within the human colonic microbiota. ISME J..

[94] Koh A., De Vadder F., Kovatcheva-Datchary P., Bäckhed F. (2016). From dietary fiber to host physiology: Short-chain fatty acids as key bacterial metabolites. Cell.

[95] La Rosa S.L., Leth M.L., Michalak L., Hansen M.E., Pudlo N.A., Glowacki R., Gabriel Pereira G., Christopher T., Workman C.T., Arntzen M.Ø. (2019). The human gut Firmicute Roseburia intestinalis is a primary degrader of dietary β-mannans. Nat. Commun..

[96] Murugesan S., Ulloa-Martínez M., Martínez-Rojano H., Galván-Rodríguez F.M., Miranda-Brito C., Romano M.C., Pina-Escobedo A., Pizano-Zárate M.L., Hoyo-Vadillo C., García-Mena J. (2015). Study of the diversity and short-chain fatty acids production by the bacterial community in overweight and obese Mexican children. Eur. J. Clin. Microbiol. Infect. Dis..

[97] Park S.K., Kim M.S., Roh S.W., Bae J.W. (2012). Blautia stercoris sp. nov., isolated from human faeces. Int. J. Syst. Evol. Microbiol..

[98] Sun M., Wu W., Liu Z., Cong Y. (2017). Microbiota metabolite short chain fatty acids, GPCR, and inflammatory bowel diseases. J. Gastroenterol..

[99] Kasahara K., Krautkramer K.A., Org E., Romano K.A., Kerby R.L., Vivas E.I., Mehrabian M., Denu J.M., Bäckhed F., Lusis A.J. (2018). Interactions between Roseburia intestinalis and diet modulate atherogenesis in a murine model. Nat. Microbiol..

[100] Atarashi K., Tanoue T., Shima T., Imaoka A., Kuwahara T., Momose Y., Cheng G., Yamasaki S., Saito T., Ohba Y. (2011). Induction of colonic regulatory T cells by indigenous Clostridium species. Science.

[101] Goverse G., Molenaar R., Macia L., Tan J., Erkelens M.N., Konijn T., Knippenberg M., Cook E.C.L., Hanekamp D., Veldhoen M. (2017). Diet-derived short chain fatty acids stimulate intestinal epithelial cells to induce mucosal tolerogenic dendritic cells. J. Immunol..

[102] Geirnaert A., Calatayud M., Grootaert C., Laukens D., Devriese S., Smagghe G., De Vos M., Boon N., Van de Wiele T. (2017). Butyrate-producing bacteria supplemented in vitro to Crohn’s disease patient microbiota increased butyrate production and enhanced intestinal epithelial barrier integrity. Sci. Rep..

[103] Nielsen D.S.G., Jensen B.B., Theil P.K., Nielsen T.S., Knudsen K.E.B., Purup S. (2018). Effect of butyrate and fermentation products on epithelial integrity in a mucus-secreting human colon cell line. J. Funct. Food.

[104] Steinmeyer S., Lee K., Jayaraman A., Alaniz R.C. (2015). Microbiota metabolite regulation of host immune homeostasis: A mechanistic missing link. Curr. Allergy Asthma. Rep..

[105] Furusawa Y., Obata Y., Fukuda S., Endo T.A., Nakato G., Takahashi D., Nakanishi Y., Uetake C., Kato K., Kato T. (2013). Commensal microbe-derived butyrate induces the differentiation of colonic regulatory T cells. Nature.

[106] Smith P.M., Howitt M.R., Panikov N., Michaud M., Gallini C.A., Bohlooly Y.M., Glickman J.N., Garrett W.S. (2013). The microbial metabolites, short-chain fatty acids, regulate colonic Treg cell homeostasis. Science.

[107] Arpaia N., Campbell C., Fan X., Dikiy S., van der Veeken J., Deroos P., Liu H., Cross J.R., Pfeffer K., Coffer P.J. (2013). Metabolites produced by commensal bacteria promote peripheral regulatory T-cell generation. Nature.

[108] Jenq R.R., Taur Y., Devlin S.M., Ponce D.M., Goldberg J.D., Ahr K.F., Littmann E.R., Ling L., Gobourne A.C., Miller L.C. (2015). Intestinal Blautia is associated with reduced death from graft-versus-host disease. Biol. Blood Marrow. Transpl..

[109] Gong J., Chehrazi-Raffle A., Placencio-Hickok V., Guan M., Hendifar A., Salgia R. (2019). The gut microbiome and response to immune checkpoint inhibitors: Preclinical and clinical strategies. Clin. Trans. Med..

[110] Cong J., Zhang X. (2018). Roles of intestinal microbiota in response to cancer immunotherapy. Eur. J. Clin. Microbiol. Infect. Dis..

[111] Chen W., Liu F., Ling Z., Tong X., Xiang C. (2012). Human intestinal lumen and mucosa-associated microbiota in patients with colorectal cancer. PLoS ONE.

[112] Bajaj J.S., Hylemon P.B., Ridlon J.M., Heuman D.M., Daita K., White M.B., Monteith P., Noble N.A., Sikaroodi M., Gillevet P.M. (2012). Colonic mucosal microbiome differs from stool microbiome in cirrhosis and hepatic encephalopathy and is linked to cognition and inflammation. Am. J. Physiol. Gastroint. Liver Physiol..

[113] Deering K.E., Devine A., O’Sullivan T.A., Lo J., Boyce M.C., Christophersen C.T. (2020). Characterizing the Composition of the Pediatric Gut Microbiome: A Systematic Review. Nutrients.

[114] Haidar Y.M., Cosman B.C. (2011). Obesity epidemiology. Clin. Colon Rectal. Surg..

[115] Festi D., Schiumerini R., Eusebi L.H., Marasco G., Taddia M., Colecchia A. (2014). Gut microbiota and metabolic syndrome. World J. Gastroenterol..

[116] Kachur S., Morera R., De Schutter A., Lavie C.J. (2018). Cardiovascular risk in patients with prehypertension and the metabolic syndrome. Curr. Hypertens Rep..

[117] Molina-Molina E., Krawczyk M., Stachowska E., Lammert F., Portincasa P. (2019). Non-alcoholic fatty liver disease in non-obese individuals: Prevalence, pathogenesis and treatment. Clin. Res. Hepatol. Gastroenterol..

[118] Eslam M., Sanyal A.J., George J. (2020). MAFLD: A consensus-driven proposed nomenclature for metabolic associated fatty liver disease. Gastroenterology.

[119] Chassaing B., Gewirtz A.T. (2014). Gut microbiota, low-grade inflammation, and metabolic syndrome. Toxicol. Pathol..

[120] D’Aversa F., Tortora A., Ianiro G., Ponziani F.R., Annicchiarico B.E., Gasbarrini A. (2013). Gut microbiota and metabolic syndrome. Intern. Emerg. Med..

[121] Natividad J.M., Agus A., Planchais J., Lamas B., Jarry A.C., Martin R., Michel M.L., Chong-Nguyen C., Roussel R., Straube S. (2018). Impaired aryl hydrocarbon receptor ligand production by the gut microbiota is a key factor in metabolic syndrome. Cell Metab..

[122] Ley R.E., Turnbaugh P.J., Klein S., Gordon J.I. (2006). Microbial ecology: Human gut microbes associated with obesity. Nature.

[123] Lippert K., Kedenko L., Antonielli L., Kedenko I., Gemeier C., Leitner M., Kautzky-Willer A., Paulweber B., Hackl E. (2017). Gut microbiota dysbiosis associated with glucose metabolism disorders and the metabolic syndrome in older adults. Benef. Microbes..

[124] Salonen A., Lahti L., Salojärvi J., Holtrop G., Korpela K., Duncan S.H., Date P., Farquharson F., Johnstone A.M., Lobley G.E. (2014). Impact of diet and individual variation on intestinal microbiota composition and fermentation products in obese men. ISME J..

[125] Zeng H., Ishaq S.L., Zhao F.Q., Wright A.D.G. (2016). Colonic inflammation accompanies an increase of β-catenin signaling and Lachnospiraceae/Streptococcaceae bacteria in the hind gut of high-fat diet-fed mice. J. Nutr. Biochem..

[126] Kostic A.D., Gevers D., Siljander H., Vatanen T., Hyötyläinen T., Hämäläinen A.M., Peet A., Tillmann V., Pöhö P., Mattila I. (2015). The dynamics of the human infant gut microbiome in development and in progression toward type 1 diabetes. Cell Host Microbe..

[127] Qin J., Li Y., Cai Z., Li S., Zhu J., Zhang F., Liang S., Zhang W., Guan Y., Shen D. (2012). A metagenome-wide association study of gut microbiota in type 2 diabetes. Nature.

[128] Kameyama K., Itoh K. (2014). Intestinal colonization by a Lachnospiraceae bacterium contributes to the development of diabetes in obese mice. Microbes Environ..

[129] Zhai B., Zhang C., Sheng Y., Zhao C., He X., Xu W., Huang K., Luo Y. (2018). Hypoglycemic and hypolipidemic effect of S-allyl-cysteine sulfoxide (alliin) in DIO mice. Sci. Rep..

[130] Schwiertz A., Taras D., Schafer K., Beijer S., Bos N.A., Donus C., Hardt P.D. (2010). Microbiota and SCFA in lean and overweight healthy subjects. Obesity.

[131] Patil D.P., Dhotre D.P., Chavan S.G., Sultan A., Jain D.S., Lanjekar V.B., Gangawani J., Shah P.S., Todkar J.S., Shah S. (2012). Molecular analysis of gut microbiota in obesity among Indian individuals. J. Biosci..

[132] Shen F., Zheng R.D., Sun X.Q., Ding W.J., Wang X.Y., Fan J.G. (2017). Gut microbiota dysbiosis in patients with non-alcoholic fatty liver disease. Hepatob. Pancreatic Dis. Int..

[133] Compare D., Coccoli P., Rocco A., Nardone O.M., De Maria S., Cartenì M., Nardone G. (2012). Gut–liver axis: The impact of gut microbiota on non alcoholic fatty liver disease. Nutr. Metab. Carbiovasc. Dis..

[134] De Minicis S., Rychlicki C., Agostinelli L., Saccomanno S., Candelaresi C., Trozzi L., Mingarelli E., Facinelli B., Magi G., Palmieri C. (2014). Dysbiosis contributes to fibrogenesis in the course of chronic liver injury in mice. Hepatology.

[135] Krawczyk M., Bonfrate L., Portincasa P. (2010). Nonalcoholic fatty liver disease. Best Pract Res. Clin. Gastroenterol..

[136] Zhu L., Baker S.S., Gill C., Liu W., Alkhouri R., Baker R.D., Gill S.R. (2013). Characterization of gut microbiomes in nonalcoholic steatohepatitis (NASH) patients: A connection between endogenous alcohol and NASH. Hepatology.

[137] Quraishi M.N., Sergeant M., Kay G., Iqbal T., Chan J., Constantinidou C., Trivedi P., Ferguson J., Adams D.H., Pallen M. (2017). The gut-adherent microbiota of PSC–IBD is distinct to that of IBD. Gut.

[138] Torres J., Bao X., Goel A., Colombel J.F., Pekow J., Jabri B., Williams K.M., Castillo A., Odin J.A., Meckel K. (2016). The features of mucosa-associated microbiota in primary sclerosing cholangitis. Aliment. Pharmacol. Ther..

[139] Chaves L.D., McSkimming D.I., Bryniarski M.A., Honan A.M., Abyad S., Thomas S.A., Wells S., Buck M., Sun Y., Genco R.J. (2018). Chronic kidney disease, uremic milieu, and its effects on gut bacterial microbiota dysbiosis. Am. J. Physiol. Renal. Physiol..

[140] De Angelis M., Montemurno E., Piccolo M., Vannini L., Lauriero G., Maranzano V., Gozzi G., Serrazanetti D., Dalfino G., Gobbetti M. (2014). Microbiota and metabolome associated with immunoglobulin A nephropathy (IgAN). PLoS ONE.

[141] Al Khodor S., Shatat I.F. (2017). Gut microbiome and kidney disease: A bidirectional relationship. Pediatr. Nephrol..

[142] Yang J., Lim S.Y., Ko Y.S., Lee H.Y., Oh S.W., Kim M.G., Cho W.Y., Jo S.K. (2018). Intestinal barrier disruption and dysregulated mucosal immunity contribute to kidney fibrosis in chronic kidney disease. Nephrol. Dial. Transplant..

[143] Wang F., Zhang P., Jiang H., Cheng S. (2012). Gut bacterial translocation contributes to microinflammation in experimental uremia. Dig. Dis. Sci..

[144] Vaziri N.D., Wong J., Pahl M., Piceno Y.M., Yuan J., DeSantis T.Z., Ni Z., Nguyen T.H., Andersen G.L. (2013). Chronic kidney disease alters intestinal microbial flora. Kidney Int..

[145] Barrios C., Beaumont M., Pallister T., Villar J., Goodrich J.K., Clark A., Pascual J., Ley R.E., Spector T.D., Bell J.T. (2015). Gut-microbiota-metabolite axis in early renal function decline. PLoS ONE.

[146] Wang Z., Klipfell E., Bennett B.J., Koeth R., Levison B.S., DuGar B., Feldstein A.E., Britt E.B., Fu X., Chung Y.M. (2011). Gut flora metabolism of phosphatidylcholine promotes cardiovascular disease. Nature.

[147] Karlsson F.H., Tremaroli V., Nookaew I., Bergström G., Behre C.J., Fagerberg B., Nielsen J., Bäckhed F. (2013). Gut metagenome in European women with normal, impaired and diabetic glucose control. Nature.

[148] Tang W.W., Wang Z., Levison B.S., Koeth R.A., Britt E.B., Fu X., Yuping Wu M.S., Hazen S.L. (2013). Intestinal microbial metabolism of phosphatidylcholine and cardiovascular risk. N. Engl. J. Med..

[149] Castillo-Rodriguez E., Fernandez-Prado R., Esteras R., Perez-Gomez M., Gracia-Iguacel C., Fernandez-Fernandez B., Kanbay M., Tejedor A., Lazaro A., Ruiz-Ortega M. (2018). Impact of altered intestinal microbiota on chronic kidney disease progression. Toxins.

[150] Hill N.R., Fatoba S.T., Oke J.L., Hirst J.A., O’Callaghan C.A., Lasserson D.S., Hobbs F.R. (2016). Global prevalence of chronic kidney disease–a systematic review and meta-analysis. PLoS ONE.

[151] Cosola C., Rocchetti M.T., Sabatino A., Fiaccadori E., Di Iorio B.R., Gesualdo L. (2019). Microbiota issue in CKD: How promising are gut-targeted approaches?. J. Nephrol..

[152] Manichanh C., Borruel N., Casellas F., Guarner F. (2012). The gut microbiota in IBD. Nat. Rev. Gastroenterol. Hepatol..

[153] Bonfrate L., Tack J., Grattagliano I., Cuomo R., Portincasa P. (2013). Microbiota in health and irritable bowel syndrome: Current knowledge, perspectives and therapeutic options. Scand. J. Gastroentero..

[154] Gevers D., Kugathasan S., Denson L.A., Vázquez-Baeza Y., Van Treuren W., Ren B., Schwager E., Knights D., Song S.J., Yassour M. (2014). The treatment-naive microbiome in new-onset Crohn’s disease. Cell Host Microbe.

[155] Baumgart M., Dogan B., Rishniw M., Weitzman G., Bosworth B., Yantiss R., Orsi R.H., Wiedmann M., McDonough P., Kim S.G. (2007). Culture independent analysis of ileal mucosa reveals a selective increase in invasive Escherichia coli of novel phylogeny relative to depletion of Clostridiales in Crohn’s disease involving the ileum. ISME J..

[156] Frank D.N., Amand A.L.S., Feldman R.A., Boedeker E.C., Harpaz N., Pace N.R. (2007). Molecular-phylogenetic characterization of microbial community imbalances in human inflammatory bowel diseases. Proc. Natl. Acad. Sci. USA.

[157] Sasaki K., Inoue J., Sasaki D., Hoshi N., Shirai T., Fukuda I., Osawa R. (2019). Construction of a model culture system of human colonic microbiota to detect decreased Lachnospiraceae abundance and butyrogenesis in the feces of ulcerative colitis patients. Biotechnol. J..

[158] Boltin D., Perets T.T., Vilkin A., Niv Y. (2013). Mucin function in inflammatory bowel disease: An update. J. Clin Gastroenterol..

[159] Torres J., Mehandru S., Colombel J.F., Peyrin-Biroulet L. (2017). Crohn’s disease. Lancet.

[160] Jellbauer S., Raffatellu M. (2014). An intestinal arsonist: Pathobiont ignites IBD and flees the scene. Gut.

[161] Duck W.L., Walter M.R., Novak J., Kelly D., Tomasi M., Cong Y., Elson C.O. (2007). Isolation of flagellated bacteria implicated in Crohn’s disease. Inflamm. Bowel. Dis..

[162] Carabotti M., Scirocco A., Maselli M.A., Severi C. (2015). The gut-brain axis: Interactions between enteric microbiota, central and enteric nervous systems. Ann. Gastroenterol..

[163] Inserra A., Rogers G.B., Licinio J., Wong M.L. (2018). The Microbiota-Inflammasome Hypothesis of Major Depression. Bioessays.

[164] Zheng P., Zeng B., Zhou C., Liu M., Fang Z., Xu X., Wang W., Tang W., Tan Z., Shi J. (2016). Gut microbiome remodeling induces depressive-like behaviors through a pathway mediated by the host’s metabolism. Mol. Psychiatr..

[165] Bonaz B., Bazin T., Pellissier S. (2018). The vagus nerve at the interface of the microbiota-gut-brain axis. Front. Neurosci..

[166] Osadchiy V., Martin C.R., Mayer E.A. (2019). The Gut–Brain Axis and the Microbiome: Mechanisms and Clinical Implications. Clin. Gastroenterol. Hepatol..

[167] Wong M.L. (2018). The inflammasome and the microbiota-gut-brain axis. Neurol. Psychiatr Brain Res..

[168] Quigley E.M. (2017). Microbiota-brain-gut axis and neurodegenerative diseases. Curr. Neurol. Neurosci. Rep..

[169] Wang X., Wang B.R., Zhang X.J., Xu Z., Ding Y.Q., Ju G. (2002). Evidences for vagus nerve in maintenance of immune balance and transmission of immune information from gut to brain in STM-infected rats. World J. Gastroenterol..

[170] Mass M., Kubera M., Leunis J.C. (2008). The gut-brain barrier in major depression: Intestinal mucosal dysfunction with an increased translocation of LPS from gram negative enterobacteria (leaky gut) plays a role in the inflammatory pathophysiology of depression. Neuroendocrinol. Lett..

[171] Cheung S., Goldenthal A.R., Uhlemann A.C., Mann J.J., Miller J.M., Sublette M.E. (2019). Systematic Review of Gut Microbiota and Major Depression. Front. Psychiatry.

[172] Jiang H., Ling Z., Zhang Y., Mao H., Ma Z., Yin Y., Wang W., Tang W., Tan Z., Shi J. (2015). Altered fecal microbiota composition in patients with major depressive disorder. Brain Behav. Immun..

[173] Berer K., Gerdes L.A., Cekanaviciute E., Jia X., Xiao L., Xia Z., Liu C., Klotz L., Stauffer U., Baranzini S.E. (2017). Gut microbiota from multiple sclerosis patients enables spontaneous autoimmune encephalomyelitis in mice. Proc. Natl. Acad. Sci. USA.

[174] Cree B.A., Spencer C.M., Varrin-Doyer M., Baranzini S.E., Zamvil S.S. (2016). Gut microbiome analysis in neuromyelitis optica reveals overabundance of Clostridium perfringens. Ann. Neurol..

[175] Cekanaviciute E., Yoo B.B., Runia T.F., Debelius J.W., Singh S., Nelson C.A., Kanner R., Bencosme Y., Kyung Lee Y., Hauser S.L. (2017). Gut bacteria from multiple sclerosis patients modulate human T cells and exacerbate symptoms in mouse models. Proc. Natl. Acad. Sci. USA.

[176] Chen J., Chia N., Kalari K.R., Yao J.Z., Novotna M., Soldan M.M.P., Luckey D.H., Marietta E.V., Jeraldo P.R., Chen X. (2016). Multiple sclerosis patients have a distinct gut microbiota compared to healthy controls. Sci. Rep..

[177] Schirmer M., Smeekens S.P., Vlamakis H., Jaeger M., Oosting M., Franzosa E.A., Horst R.T., Jansen T., Jacobs L., Bonder M.J. (2016). Linking the Human Gut Microbiome to Inflammatory Cytokine Production Capacity. Cell.

[178] Shahi S.K., Freedman S.N., Mangalam A.K. (2017). Gut microbiome in multiple sclerosis: The players involved and the roles they play. Gut Microbes.

[179] Cekanaviciute E., Debelius J.W., Singh S., Runia T., Nelson C., Yoo B. Gut dysbiosis is a feature of MS and it is characterized by bacteria able to regulate lymphocyte differentiation in vitro. Proceedings of the 2016 European Committee for Treatment and Research in Multiple Sclerosis.

[180] Jangi S., Gandhi R., Cox L.M., Li N., von Glehn F., Yan R., Patel B., Mazzola M.A., Liu S., Glanz B.L. (2016). Alterations of the human gut microbiome in multiple sclerosis. Nat. Commun..

[181] Bibbò S., Ianiro G., Giorgio V., Scaldaferri F., Masucci L., Gasbarrini A., Cammarota G. (2016). The role of diet on gut microbiota composition. Eur. Rev. Med. Pharmacol. Sci..

[182] Flint H.J., Duncan S.H., Scott K.P., Louis P. (2015). Links between diet, gut microbiota composition and gut metabolism. Proc. Nutr. Soc..

[183] Scott K.P., Gratz S.W., Sheridan P.O., Flint H.J., Duncan S.H. (2013). The influence of diet on the gut microbiota. Pharmacol. Res..

[184] Wu G.D., Chen J., Hoffmann C., Bittinger K., Chen Y.Y., Keilbaugh S.A., Bewtra M., Knights D., Walters W.A., Knight R. (2011). Linking long-term dietary patterns with gut microbial enterotypes. Science.

[185] De Angelis M., Ferrocino I., Calabrese F.M., De Filippis F., Cavallo N., Siragusa S., Rampelli S., Di Cagno R., Rantsiou K., Vannini L. (2020). Diet influences the functions of the human intestinal microbiome. Sci. Rep..

[186] Rinninella E., Cintoni M., Raoul P., Lopetuso L.R., Scaldaferri F., Pulcini G., Miggiano G.A.D., Gasbarrini A., Mele M.C. (2019). Food components and dietary habits: Keys for a healthy gut microbiota composition. Nutrients.

[187] Flint H.J., Scott K.P., Louis P., Duncan S.H. (2012). The role of the gut microbiota in nutrition and health. Nat. Rev. Gastroenterol. Hepatol..

[188] Wilson M., Nibali L., Henderson B. (2009). Bacteriology of Humans: An Ecological Perspective.

[189] Tottey W., Feria-Gervasio D., Gaci N., Laillet B., Pujos E., Martin J.F., Sebedio J.L., Sion B., Jarrige J.F., Alric M. (2017). Colonic transit time is a driven force of the gut microbiota composition and metabolism: In vitro evidence. J. Neurogastroenterol. Motil..

[190] Flint H.J., Duncan S.H., Scott K.P., Louis P. (2007). Interactions and competition within the microbial community of the human colon: Links between diet and health. Environ. Microbiol..

[191] Belenguer A., Duncan S.H., Calder A.G., Holtrop G., Louis P., Lobley G.E., Flint H.J. (2006). Two routes of metabolic cross-feeding between Bifidobacterium adolescentis and butyrate-producing anaerobes from the human gut. Appl. Environ. Microbiol..

[192] Aminov R.I., Walker A.W., Duncan S.H., Harmsen H.J.M., Welling G.W., Flint H.J. (2006). Molecular diversity, cultivation and improved detection by fluorescent in situ hybridization of a dominant group of human gut bacteria related to Roseburia spp. or Eubacterium rectale. Appl. Environ. Microbiol..

[193] Martínez I., Lattimer J.M., Hubach K.L., Case J.A., Yang J., Weber C.G., Louk J.A., Rose D.J., Kyureghian G., Peterson D.A. (2013). Gut microbiome composition is linked to whole grain-induced immunological improvements. ISME J..

[194] Di Iorio B.R., Rocchetti M.T., De Angelis M., Cosola C., Marzocco S., Di Micco L., di Bari I., Accetturo M., Vacca M., Gobbetti M. (2019). Nutritional Therapy Modulates Intestinal Microbiota and Reduces Serum Levels of Total and Free Indoxyl Sulfate and P-Cresyl Sulfate in Chronic Kidney Disease (Medika Study). J. Clin. Med..

[195] De Filippis F., Pellegrini N., Vannini L., Jeffery I.B., La Storia A., Laghi L., Serrazanetti D.I., Di Cagno R., Ferrocino I., Lazzi C. (2016). High-level adherence to a Mediterranean diet beneficially impacts the gut microbiota and associated metabolome. Gut.

[196] Daniel H., Gholami A.M., Berry D., Desmarchelier C., Hahne H., Loh G., Mondot S., Lepage P., Rothballer M., Walker A. (2014). High-fat diet alters gut microbiota physiology in mice. ISME J..

[197] Ortega J.F., Morales-Palomo F., Fernandez-Elias V., Hamouti N., Bernardo F.J., Martin-Doimeadios R.C., Nelson R.K., Horowitz J.F., Mora-Rodriguez R. (2016). Dietary supplementation with omega-3 fatty acids and oleate enhances exercise training effects in patients with metabolic syndrome. Obesity.

[198] Marik P.E., Varon J. (2009). Omega-3 dietary supplements and the risk of cardiovascular events: A systematic review. Clin. Cardiol..

[199] Liu J., Ma D.W. (2014). The role of n-3 polyunsaturated fatty acids in the prevention and treatment of breast cancer. Nutrients.

[200] Noriega B.S., Sanchez-Gonzalez M.A., Salyakina D., Coffman J. (2016). Understanding the impact of omega-3 rich diet on the gut microbiota. Case Rep. Med..

[201] Menni C., Zierer J., Pallister T., Jackson M.A., Long T., Mohney R.P., Steves C.J., Spector T.D., Valdes A.M. (2017). Omega-3 fatty acids correlate with gut microbiome diversity and production of N-carbamylglutamate in middle aged and elderly women. Sci. Rep..

[202] Howe P., Buckley J. (2014). Metabolic health benefits of long-chain omega-3 polyunsaturated fatty acids. Mil. Med..

[203] Horrocks L.A., Yeo Y.K. (1999). Health benefits of docosahexaenoic acid (DHA). Pharmacol. Res..

[204] Kim E., Kim D.B., Park J.Y. (2016). Changes of mouse gut microbiota diversity and composition by modulating dietary protein and carbohydrate contents: A pilot study. Prev. Nutr. Food Sci..

